# Successful Management of Hemodynamically Unstable Takotsubo Cardiomyopathy With Milrinone

**DOI:** 10.7759/cureus.27820

**Published:** 2022-08-09

**Authors:** Karan H Pahuja, Natale Wasef, Syed Hasan, Tehreem Fatima, Steven Hamilton, Marc Seelagy

**Affiliations:** 1 Internal Medicine, Jersey Shore University Medical Center/Saint Francis Medical Center Program, Trenton, USA

**Keywords:** takotsubo cardiomyopathy (ttc), stress-induced cardiomyopathy, cardiogenic shock, inotropes, milrinone

## Abstract

Takotsubo cardiomyopathy (TTC) was initially reported in the 1990s as a reversible cause of cardiomyopathy induced by acute emotional stress. It is characterized by regional systolic dysfunction in the absence of coronary artery disease. We report a case of a 79-year-old woman who was admitted with acute respiratory failure due to pneumonia and was found to have a troponin elevation. Upon further evaluation, the patient was taken to the cardiac catheterization lab and underwent catheterization which showed apical ballooning concerning Takotsubo cardiomyopathy. She was placed on a norepinephrine drip but remained unstable. Milrinone-facilitated diuresis was then initiated with improvement and stabilization in hemodynamics. Takotsubo cardiomyopathy presenting with cardiogenic shock without left ventricular outflow tract obstruction requires treatment with inotropes. Although there is limited data to support the use of milrinone in cardiogenic shock due to TTC, its use in our case facilitated diuresis and improved the patient’s outcome after norepinephrine failed to stabilize our patient’s hemodynamics. Milrinone inhibits phosphodiesterase type 3 which increases the calcium influx thereby improving the myocardial contraction without any beta agonist action. Therefore, the use of milrinone which is a non-catecholamine inotrope could be considered a better alternative as compared to dobutamine given the underlying pathophysiology of TTC.

## Introduction

Takotsubo cardiomyopathy (TTC) was first reported in the 1990s by Japanese authors. It is reversible cardiomyopathy that is precipitated by acute emotional stress. TTC is characterized by transient regional systolic dysfunction of the left ventricle in the absence of coronary artery disease or acute plaque rupture. In most Takotsubo cardiomyopathy cases, a large area of the heart wall is involved, which is perfused by multiple coronary arteries [1]. TTC is also known as transient apical ballooning syndrome, stress-induced cardiomyopathy, stress cardiomyopathy, and broken-heart syndrome. It is much more common in women than men and occurs predominantly in older individuals [2]. Various hypotheses have been proposed to understand the possible pathophysiological mechanisms. The catecholamine hypothesis is widely accepted. According to this hypothesis, stress, estrogen deficiency, microvascular dysfunction, or microcirculatory disorder could lead to disproportionate catecholamine secretion, which can stun the myocardium [3]. There is no data available regarding ideal treatment of Takotsubo cardiomyopathy, but therapy is guided by the patient’s clinical presentation and hemodynamic status. In stable patients, treatment modalities include cardio-selective beta-blockers and angiotensin-converting enzyme (ACE) inhibitors. Patients with unstable hemodynamics (hypotension) may be treated with inotropes [4]. Here, we present a case of Takotsubo cardiomyopathy that was successfully treated with milrinone. 

This article was previously presented as a poster abstract at the 2021 American College of Cardiology (ACC) Latin America Conference on November 5, 2021.

## Case presentation

A 79-year-old woman was brought to our emergency department after being intubated in the field by the EMS due to worsening shortness of breath and productive cough that started earlier that day. The nasotracheal route was used after failed attempts at the oropharyngeal route. 

Her past medical history was significant for coronary artery disease (CAD) status post three drug-eluting stents and heart failure with mid-range ejection fraction (HFmrEF) with ejection fraction (EF) 40-45%, systemic hypertension, prediabetes, obesity, and gastroesophageal reflux disease (GERD). On examination, the patient had 1+ bilateral lower extremity pitting edema, predominantly right-sided rhonchi, without prominent jugular venous distension (JVD) or a third heart sound (S3). Laboratory values are shown in Table 1.

**Table 1:** Laboratory values of the patient PaCO_2_: partial pressure of carbon dioxide; BNP: brain natriuretic peptide; BUN: blood urea nitrogen; WBC: white blood cell; RT-PCR: reverse transcriptase polymerase chain reaction

Test	Results	Reference	Unit
pH	7.15	7.35-7.45	-
Arterial PaCO_2_	91.3	35-45	mmHg
Arterial bicarbonate	33.8	22-26	mmol/L
BNP	750	<100	pg/mL
Creatinine	1.2	0.49-1.01	mg/dL
BUN	22	7-25	mg/dL
Lactate	2.8	0.5-2.0	mmol/L
WBC count	10.9	3.8-10.2	10^3^/uL
Neutrophil count	61	42.7-76.7	%
COVID-19 RT-PCR test	Negative	Negative	-

Chest x-ray (CXR) demonstrated bilateral, right greater than left, opacities, and left costophrenic angle blunting which was suggestive of pulmonary edema and/or pneumonia. She received a one-time dose of furosemide 60mg intravenously (IV) with minimal urine output. Additionally, she was initiated on ceftriaxone, azithromycin, and metronidazole. 

She required minimal settings on the ventilator, however, failed a spontaneous breathing trial on day 2 due to tachypnea. Over the next two days, her ventilator requirements increased from a positive end-expiratory pressure (PEEP) of 5 to a PEEP of 10. Additionally, the patient went into new-onset atrial fibrillation with controlled ventricular response (CVR) and was subsequently started on IV unfractionated heparin. 

**Figure 1:**
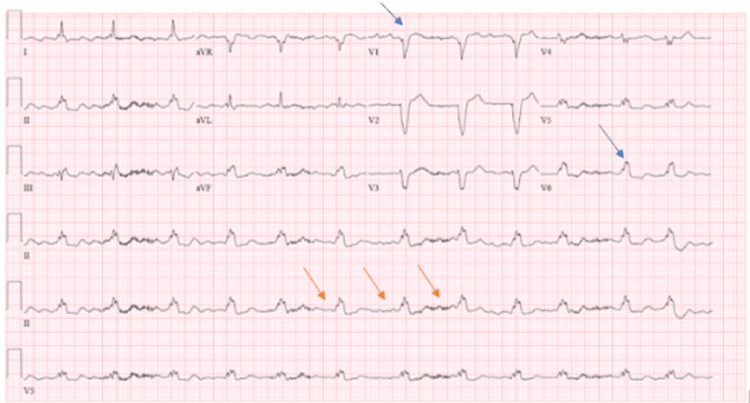
EKG showing atrial fibrillation with controlled ventricular response during the stress-induced cardiomyopathy event Blue arrows showing the left bundle branch block and orange arrows showing the irregular rhythm and absent p-wave consistent with atrial fibrillation.

Spontaneous breathing trial was successful on day 5 of admission and she was extubated via her nasopharyngeal passage. Upon extubation, the patient was noted to have significant epistaxis. Her IV unfractionated heparin was immediately discontinued and the nasal passage was packed with epinephrine-soaked gauze. Subsequently, she endorsed a sense of doom and severe substernal chest pain. Electrocardiogram (EKG) showed atrial fibrillation with CVR and chronic left bundle branch block (Figure 1).

**Figure 2:**
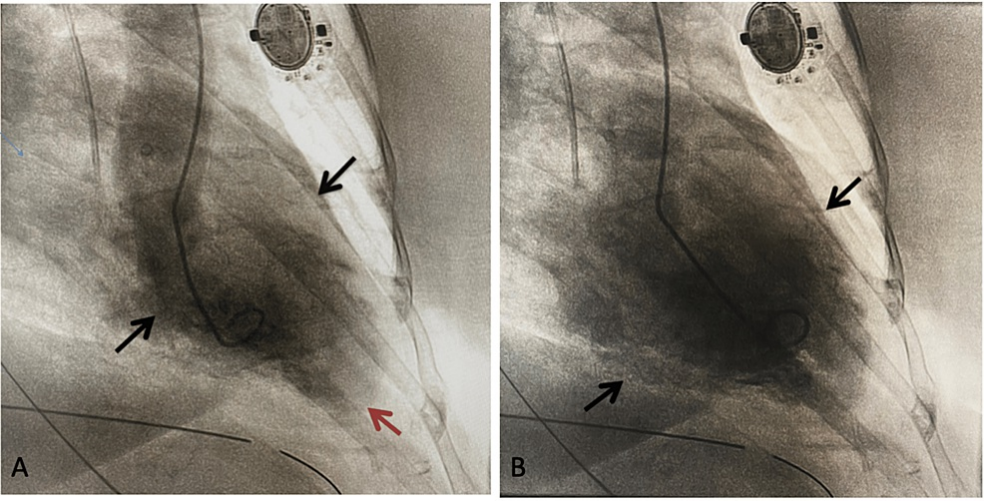
Invasive coronary angiogram demonstrating Takotsubo pattern (A) Systole - red arrow showing apical akinesia and black arrows showing normokinetic basal segment; (B) diastole - black arrows showing normokinetic basal segment.

Troponin level was 2.4 ng/mL, increased from 0.06 ng/mL on admission and she became increasingly tachypneic requiring reintubation via the orotracheal route. Immediate cardiac catheterization revealed apical and mid segments akinesia with normokinetic basal segments consistent with Takotsubo cardiomyopathy (Figures 2A, 2B). 

**Figure 3:**
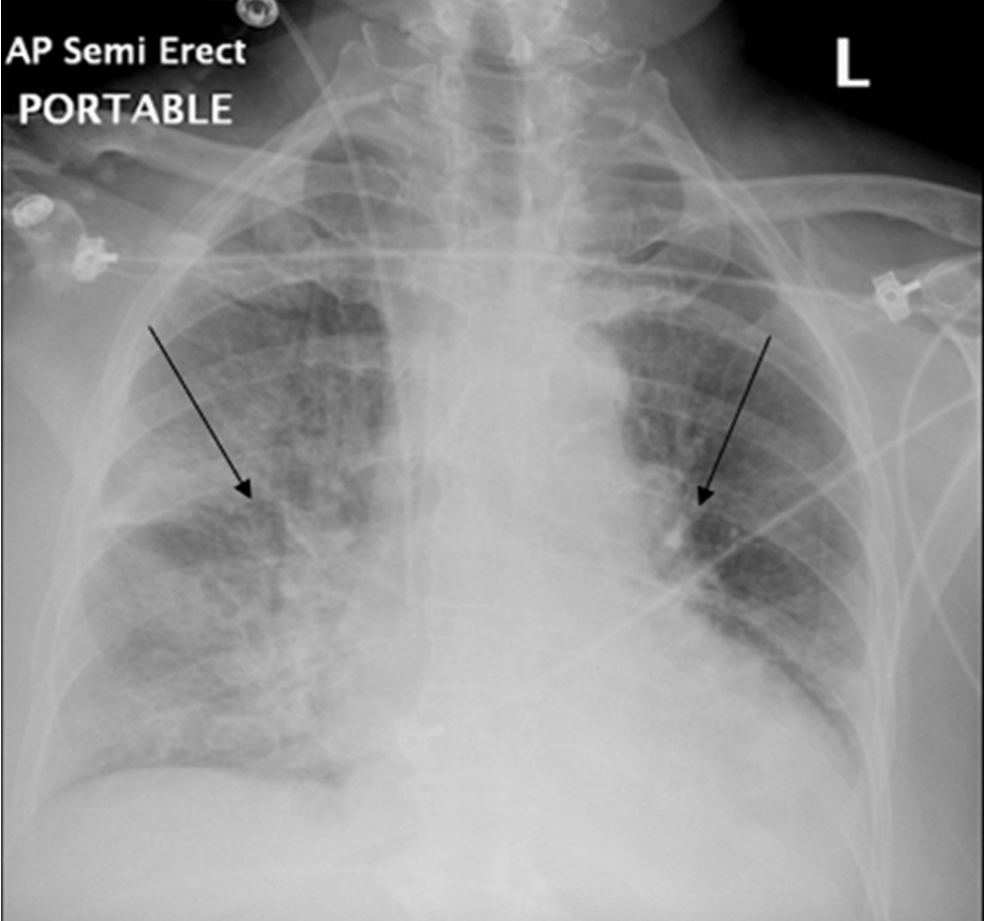
Chest x-ray showing with the black arrows showing vascular congestion/pulmonary edema

There was mild non-obstructive CAD with patent stents and left ventricular (LV) end-diastolic pressure of 16 mmHg. Additionally, a transthoracic echocardiogram revealed severely reduced left ventricular systolic function, EF 20-25%, an aneurysmal LV anterior wall and multiple wall motion abnormalities, and no LV outflow tract obstruction. The patient was started on IV amiodarone after which she converted to sinus rhythm and was restarted on IV unfractionated heparin since her hemoglobin remained stable and there was no recurrence of the epistaxis. Within 24 hours, there were multiple episodes of hypotension and vascular congestion on chest x-ray, which required the use of norepinephrine, with hemodynamics remaining labile (Figure 3).

On day 11, she was successfully extubated again to room air and remained comfortable throughout the day. Unfortunately, she became tachypneic and restless overnight, requiring reintubation for the third time. After the patient’s third reintubation, her blood pressure remained labile to the point where the furosemide had to be held, however, the following day her central venous pressure (CVP) increased to 23 mmHg. Since diuresis was limited due to persistent hypotension, milrinone-assisted diuresis was initiated at 0.25 mcg/kg/min. After three days of milrinone-assisted diuresis, the patient was aggressively diuresed with furosemide 40 mg IV twice daily to euvolemia with hemodynamic stability. On day 19 of admission, the patient was successfully extubated to bi-level positive airway pressure. Her oxygen requirements decreased over the next few days and the milrinone was eventually discontinued. The patient did well off the milrinone and was maintained on oral diuretics and nasal cannula until discharge. She will have a repeat transthoracic echocardiogram in 90 days.

## Discussion

Patients with Takotsubo cardiomyopathy (TTC) presenting with cardiogenic shock without left ventricular outflow tract obstruction require treatment with inotropes. Cardiogenic shock is most frequently treated with dobutamine or epinephrine [5,6]. However, there is little data available on the use of milrinone in the case of cardiogenic shock due to TTC.

There is no optimal medical regimen for stress-induced cardiomyopathy. The driving pathophysiology is disproportionate catecholamine release leading to direct myocardial injury and stunning. Although it is a transient disorder that requires supportive therapy, some patients like ours develop acute complications like shock and acute heart failure. Given that TTC is related to disproportionate catecholamine release which consequently stuns the myocardium, use of milrinone, a non-catecholamine inotrope is a reasonable option instead of catecholamine inotropes [7]. 

Takotsubo cardiomyopathy with hypotension and heart failure is an ICU dilemma for which the treatment is debatable. Catecholamine inopressors such as norepinephrine may cause paradoxical beta receptor negative inotropic effects, necessitating the use of milrinone. Milrinone inhibits phosphodiesterase type 3 and increases the calcium influx thereby improving the myocardial contraction without any beta agonist action [8]. There is also an added advantage of less increase in the heart rate and myocardial oxygen demand as compared to dobutamine. However, it may cause hypotension due to peripheral vasodilation [8]. 

Very few cases have been reported regarding the use of milrinone in cases of cardiogenic shock due to TTC. In our case, milrinone was chosen considering the underlying pathophysiology of TTC and the risk of arrhythmias with dobutamine given that this patient had new-onset atrial fibrillation during hospitalization. Doyen et al. reported one of the initial cases of TTC where lactate levels and cardiac index improved only after starting milrinone infusion [7]. Mrozek et al. also reported a case where dobutamine infusion was discontinued in TTC due to tachyarrhythmia followed by milrinone which stabilized the patient's hemodynamic status and improved cardiac output without deleterious effects [9]. Milrinone has also shown benefits in acute right heart failure due to isolated right ventricular Takotsubo syndrome [10]. To further support the use of a non-catecholamine inotrope, dobutamine infusion is known to trigger TTC [11,12]. Another non-catecholamine inotrope such as levosimendan has also shown successful outcomes in the treatment of TTC [13]. However, it is not commonly used given the increased risk of vasoplegia and hypotension [14]. The recent Dobutamine Compared with Milrinone (DOREMI) trial showed that, among patients with cardiogenic shock, there were no significant differences in cardiovascular or renal outcomes between intravenous milrinone and dobutamine [15]. However, when it comes to cardiogenic shock due to Takotsubo cardiomyopathy the use of a non-catecholamine inotrope like milrinone could be considered a better alternative as compared to dobutamine given the underlying pathophysiology of TTC.

## Conclusions

Takotsubo cardiomyopathy with hypotension and heart failure is an ICU dilemma. Catecholamine inopressors may cause negative inotropic effects, necessitating use of milrinone. It provides afterload reduction, positive inotropy, less heart rate increase, and less myocardial oxygen consumption. Ultimately, this facilitates effective diuresis.
